# A Comparative Study on β‐ODAP Level, Polyphenolic Content and Antioxidant Activity Across Three *Lathyrus sativus* L. Cultivars

**DOI:** 10.1002/fsn3.72355

**Published:** 2026-09-09

**Authors:** Giulia Gentile, Lorenza Marinaccio, Eleonora Procino, Angelo Cichelli, Adriano Mollica, Azzurra Stefanucci

**Affiliations:** ^1^ Department of Pharmacy University of Chieti‐Pescara “G. d'Annunzio” Chieti Italy; ^2^ Department of Innovative Technologies in Medicine and Dentistry University “G. d'Annunzio” of Chieti‐Pescara Chieti Italy; ^3^ SCM Nutraceutici Universitari Srl Chieti Italy

**Keywords:** β‐ODAP, anti‐nutrients, antioxidant activity, neurolathyrism, polyphenols

## Abstract

Grass pea ( *Lathyrus sativus* L.) is an old crop highly resistant to climate changes and drought. Despite its nutritional benefits, it represents a source of β‐ODAP, a non‐protein amino acid which causes neurolathyrism. This study aims to determine the β‐ODAP content of three different Italian cultivars of grass pea collected in Umbria and Puglia. An aqueous extraction of each grass pea sample was carried out and analyzed by High Performance Liquid Chromatography (HPLC) with a Diode Array Detector (DAD) after Fmoc‐derivatization to quantify β‐ODAP. Furthermore, the polyphenolic content and antioxidant activity of each grass pea sample have been evaluated by performing spectrophotometric analysis. The β‐ODAP content ranged from 0.01 mg/g to 0.037 mg/g of grass pea powder, resulting in being higher in the biological cultivar. Sample C (Puglia grass pea) showed the best antioxidant activity. Overall, these results encourage the cultivation of this crop and prompt further studies on the biofunctional properties of this resistant legume.

## Graphical abstract

β‐ODAP content in three diverse *Lathyrus sativus* L. cultivars.

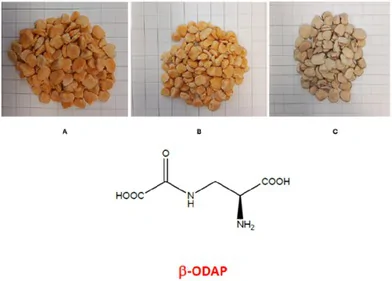

## 1 Introduction

Grass pea ( *Lathyrus sativus* L.) belongs to the family of *Fabaceae* (*Leguminosae*). This legume is highly resistant to climate change and to drought conditions; so as it is commonly known as a “survival food”, easily available in dry seasons and at low prices (Lambein et al. 2019; Miah et al. 2024).

The symbiotic relationship with *Rhizobia* makes this plant even more interesting; indeed, *Rhizobia* is a bacteria able to fix atmospheric nitrogen in the soil, transforming nitrogen gas into chemical compounds which can be absorbed and used for the synthesis of macromolecules (e.g., nucleic acids and proteins) by plants. In this way the application of fertilizers is unnecessary resulting in being economically advantageous and convenient in terms of annual crop rotation (Asrat et al. 2023; Kebede 2021).

Grass pea represents one of the most ancient crops; it is cultivated in Europe, Asia, and Africa and used for cereal supplementation, livestock feed, and human consumption (Gentile et al. 2025; Lambein et al. 2019; Miah et al. 2024). This cultivation, recognized as an “orphan legume crop” is underrated in comparison with other commercial crops, but it has a significant value in terms of nutritional benefits and economical aspects. Actually, grass pea is rich in proteins (18%–34%) and total fatty acids lower than 2% (58% of which are polyunsaturated fatty acids) (Simões et al. 2023).

The proteins contained in the seeds comprise globulins (66%), glutelins (15%), albumins (14%) and prolamins (5%) (Chandna and Matta 1994; Lambein et al. 2019). In terms of amino acid profile, as the majority of grain legumes, grass pea contains low quantities of methionine and cysteine (Iqbal et al. 2006). On the contrary, it is rich in lysine, generally low in cereals, which is an essential amino acid crucial in protein synthesis and in human nutrition (Matthews 2020).

Moreover, grass pea is a dietary source of L‐homoarginine (hArg), becoming an example of potential functional food (Lambein et al. 2019; Llorent‐Martínez et al. 2017; Singh and Rao 2013). Recent studies reported the correlation between low levels of hArg and the increased risk of cardiovascular disease (Karetnikova et al. 2019).

**FIGURE 1:**
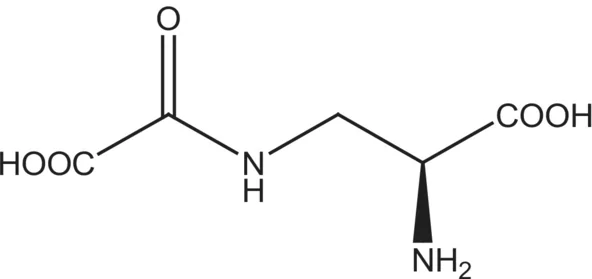
Structure of β‐ODAP.

On the other hand, grass pea is also known as a source of anti‐nutritional factors and ODAP (N‐oxalyl‐L‐α,β‐diaminopropionic acid) (Urga et al. 2012). The non‐protein amino acid β‐ODAP (β‐N‐oxalyl‐L‐α,β‐diaminopropionic acid) is a neurotoxin responsible for the onset of neurolathyrism, a neurological disorder (Simões et al. 2023) (Figure 1).

In humans, neurolathyrism causes spastic paraparesis which affects mainly the Betz cells as well as the corticospinal tracts (Ludolph and Spencer 1996; Ravindranath 2002). The β‐ODAP content in *Lathyrus* seeds is < 0.2 g/100 g; the overconsumption of grass pea can lead to β‐ODAP accumulation in the central nervous system causing convulsions (Miah et al. 2024).

While the anti‐nutritional factor cannot be eliminated, it is sufficient to soak and cook the grass pea to reduce its concentration (Linee guida per una sana alimentazione 2017). Different techniques have been reported in literature to determine the content of β‐ODAP, for example, spectrophotometric, mass spectrometric, HPLC methods (Emmrich et al. 2019; Miah et al. 2024). Many researchers apply a pre‐column derivatization to analyze amino acids, using different derivatization groups such as 9‐fluorenylmethyl chloroformate (Fmoc‐Cl), ophthalaldehyde (OPA), and dansyl chloride, etc. (Jámbor and Molnár‐Perl 2009; Pencheva et al. 2022; Thippeswamy et al. 2007). Some of these cannot differentiate β‐ODAP from its isomer α‐ODAP, naturally present in < 5% amount, and it is not neurotoxic.

Besides the β‐ODAP content of grass pea, this legume has also been investigated for its polyphenols composition and antioxidant properties (Rybiński et al. 2018; Starzyńska‐Janiszewska et al. 2008). Indeed, polyphenols are gaining attention in the last decades for their health promoting effects due to their antioxidant properties (Pastor‐Cavada et al. 2009).

Considering the growing interest for such resistant and ancient crop, in this work the content of β‐ODAP of three diverse grass pea cultivars has been determined applying a well‐established pre‐derivatization procedure with Fmoc‐Cl following HPLC‐DAD technique, with the aim to investigate the influence of different soil composition in amino acid enrichment. We selected three types of samples, specifically, two varieties of decorticated grass pea belonging to non‐biological crops from Umbria and Puglia and a decorticated grass pea belonging to a biological crop from Umbria, due to their easy availability in the regional local market and the optimum price‐quality ratio. Furthermore, these are among few Italian regions that still cultivate this legume, for which a traditional history of culinary consumption is well known. A comparative analysis was carried out to determine the difference in terms of total phenolic content (TPC), total flavonoid content (TFC), and antioxidant properties among the different grass pea samples.

## 2 Materials and Methods

### 2.1 Reagents and Materials

β‐ODAP ≥ 98% (HPLC), Fmoc chloride (for HPLC derivatization, ≥ 99.0%), Sodium acetate anhydrous (≥ 99.0%), Gallic acid monohydrate (≥ 99% HPLC), Sodium carbonate powder (≥ 99.5%, ACS reagent), Potassium Persulfate (99.99% trace metals basis), Iron(III) chloride hexahydrate (ACS reagent, 97%), 2,2′‐azino‐bis(3‐ethylbenzothiazoline‐6‐solfonic acid) diammonium salt (≥ 98% (HPLC), chromogenic, powder), (±)‐6‐hydroxy‐2,5,7,8‐tetra‐methylchromane‐2‐carboxylic acid (97%), 2,4,6‐Tris(2‐pyridyl)‐*s*‐triazine (for spectrophotometric det. of Fe, ≥ 99.0% HPLC), 2,2‐Diphenyl‐1‐picrylhydrazyl (powder), Acetic Acid (glacial, ACS reagent, ≥ 99.7%) Water (HPLC Plus), Acetonitrile (≥ 99.9%, HPLC), Ethanol puriss. 96.0%–97.2%, Ethyl acetate puriss. p.a. ACS reagent ≥ 99.5% (GC), Hexane puriss. p.a. ACS reagent ≥ 99% (GC), Acetone (ACS reagent, ≥ 99.5%) were all purchased from Sigma‐Aldrich (Milan, Italy). Folin‐Ciocalteau's reagent (for analysis of phenols) and Methanol (Reag. Ph. Eur. for HPLC—gradient grade) were purchased from VWR Chemicals BDH (Milan, Italy). Rutin trihydrate was purchased from Santa Cruz Biotechnology (Heidelberg, Germany). Aluminum chloride puriss. p.a., anhydrous, crystallized, ≥ 99.0% (AT) was purchased from Honeywell Fluka (Segrate, Italy).

A Rotavapor R‐80 BUCHI (Cornaredo, Italy) was used for solvent evaporation. An Agilent Technologies 1100 Series with ALS (G1313A), Degasser (G1379A), Bin Pump (G1312A), Col Comp (G1316A), and DAD (G1315C) was used for the analysis (Santa Clara, CA, United States). A Jasco V‐730 UV–Vis Spectrophotometer (Cremella, Italy) was used for the determination of TPC and TFC and to perform the antioxidant assays.

### 2.2 Matrix and Sample Preparation

**FIGURE 2:**
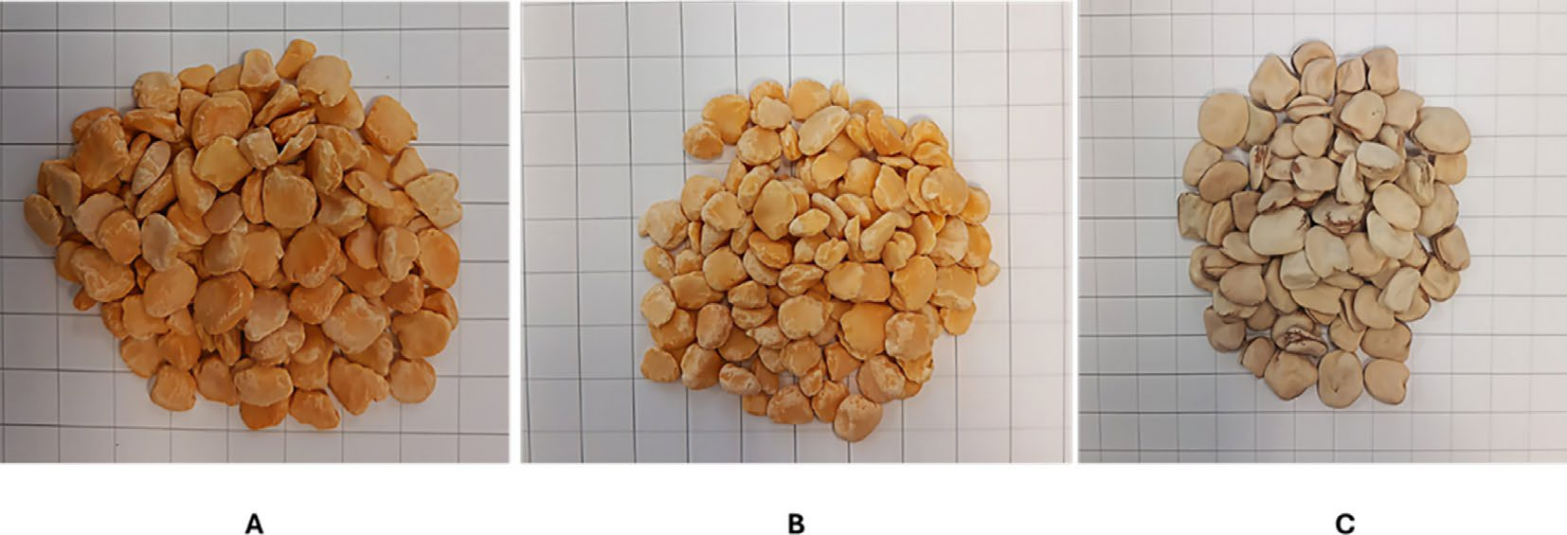
Diverse grass peas examined in this study: (A) Umbria grass pea belonging to non‐biological crop, (B) Umbria grass pea belonging to a biological crop, (C) Puglia grass pea belonging to a non‐biological crop.

In this study, two varieties of *Lathyrus sativus* L. seeds (decorticated grass pea) belonging to a non‐biological and a biological crop cultivated in the area of Colfiorito (Foligno, Umbria, Italy) and a non‐biological decorticated grass pea cultivated in the Murgia Barese area (Bari, Puglia, Italy) have been purchased from local farmers. The biological crop was cultivated according to the organic farming regulations, which include restrictions on the application of fertilizers and other agricultural practices. The non‐biological crops were cultivated following the conventional agricultural practices, allowing the use of authorized fertilizers and plant protection products. For the sake of clarity, each sample has been renamed as sample A (Umbria grass pea from a non‐biological crop), sample B (Umbria grass pea from a biological crop), and sample C (Puglia grass pea from a non‐biological crop) as described in Figure 2. The three samples of grass pea were triturated in a blender and lyophilized. The decorticated grass pea powders were stored at −20°C until their extraction.

### 2.3 Extraction Procedure

Approximately 500 mg of decorticated grass pea powder (503.5 mg of sample A; 500.7 mg of sample B; 505.1 mg of sample C) was mixed with 3 mL of water and stirred at room temperature for 1 h. The solution was centrifuged at 4400 rpm for 20 min and then filtered through a 0.45 μm diameter filter. Finally, the filtered solution was lyophilized. The final yield was expressed as:

$$\left(\frac{\mathrm{mg}\;\text{extract}}{\mathrm{mg}\;\text{grass}\;\mathrm{pea}\;\text{powder}}\right)\times 100$$

### 2.4 Derivatization of the β‐ODAP Analytical Standard and Grass Pea Extracts

**FIGURE 3:**
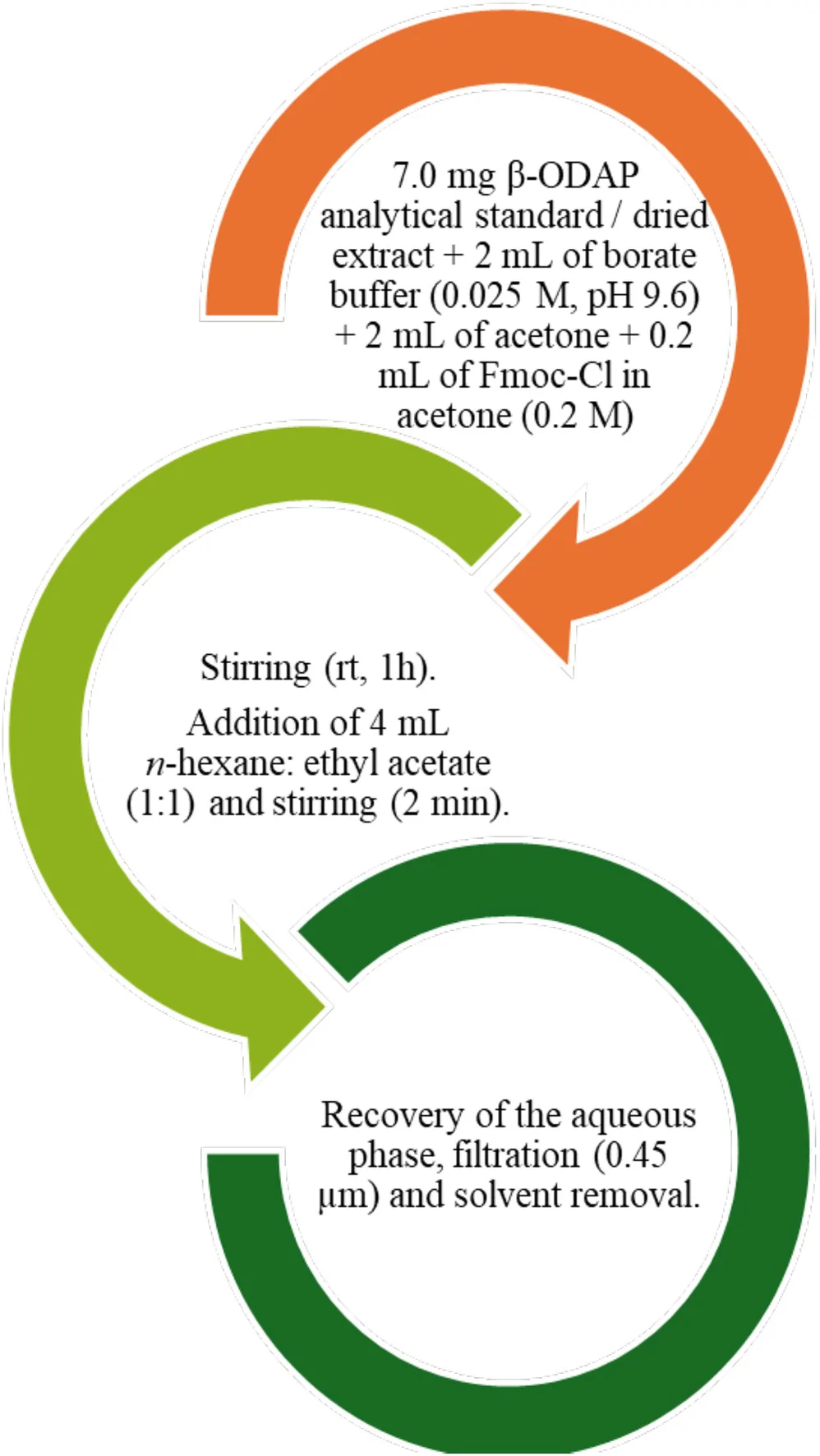
Workflow of β‐ODAP derivatization.

To derivatize the analytical standard and the samples, the method reported by Geda et al. was followed with slight modifications (Figure 3) (Geda et al. 1993).

A solution of Fmoc‐Cl in acetone (0.2 M) was freshly prepared prior each sample derivatization. β‐ODAP analytical standard or the dried extract (7.0 mg) was mixed with 2 mL of borate buffer (0.025 M, pH 9.6), 2 mL of acetone and 0.2 mL of Fmoc‐Cl in acetone (0.2 M). The solution was stirred at room temperature for 1 h. Then, 4 mL of *n*‐hexane: ethyl acetate (1:1) was added and stirred for 2 min. The aqueous phase was separated from the organic one and filtered by a 0.45 μm filter. After solvent removal by rotary evaporation and high vacuum (1.5 h), the Fmoc‐derivatized dried product was conserved at −20°C until the analysis.

### 2.5 HPLC Analysis

The analyses have been performed by using a HPLC‐DAD (Agilent 1100 Series) with a reversed‐phase C18 analytical column (5 μm particle size, 150 × 3.0 mm). The HPLC analyses were carried out following Geda et al. in isocratic conditions using as mobile phase sodium acetate (0.05 M, pH 3)/acetonitrile (72:28 *v/v*) (Geda et al. 1993). The flow rate was set to 1 mL/min, the injection volume was 2 μL, the column temperature was maintained at 23°C. The analysis was run for 10 min. The determination and quantification of β‐ODAP were performed at 254 nm after its Fmoc‐derivatization. The calibration graph was obtained plotting the area vs the concentration (mg L^−1^) considering a range between 5 and 1000 mg L^−1^. The calibration graph had a *R* ^2^ > 0.998. After each analysis, 100% acetonitrile was used to flush the column and a re‐equilibration with the mobile phase was performed before the injection of the next sample. Each Fmoc‐derivatised extract was redissolved in water (1 mg/mL) to perform the HPLC analyses.

### 2.6 Folin Ciocalteu Assay

The determination of TPC was performed by applying the Folin–Ciocalteu assay following Singleton & Rossi with slight modifications (Raposo et al. 2024; Singleton and Rossi 1965). In brief, 0.25 mL of extract (solubilized in H_2_O) was mixed with 1.25 mL of Folin reagent diluted 1:10. After 5 min (room temperature in the dark), 1 mL of Na_2_CO_3_ 7.5% (*p/v*) was added. The samples were incubated for 90 min at room temperature in dark conditions, and the absorbance was read at 760 nm. The assay was performed in triplicate for each extract. A calibration curve using gallic acid as an external standard was prepared in a concentration range of 5–100 mg L^−1^ (*y* = 0.0106*x* + 0.0109, *R* ^2^ = 0,9997). The results were expressed as mg GAE/g DW (GAE: gallic acid equivalents; DW: dry weight of grass pea).

### 2.7 Aluminum Chloride Assay

The evaluation of the TFC was conducted by performing the aluminum chloride assay (Arvouet‐Grand et al. 1994). The dried extracts were resolubilized in a mixture of MeOH:H_2_O 80:20, sonicated, and filtered (0.45 μm). The sample (1 mL) was mixed with a solution of AlCl_3_ 2% in methanol (1 mL) and incubated for 30 min at room temperature in the dark. The absorbance of each sample was read at 415 nm. A sample blank (sample mixed with methanol without AlCl_3_) was necessary to correct the sample absorbance. A calibration curve was prepared using rutin as an external standard (5–75 mg L^−1^) (*y* = 0.0149*x* − 0.0326, *R* ^2^ = 0,9993). The results, expressed as mg RE/g DW (RE: rutin equivalents), are the media of three experiments.

### 2.8 DPPH Assay

The DPPH assay was performed following the procedure reported by Zengin & Aktumsek with slight modifications (Zengin and Aktumsek 2014). The extracts were solubilized in MeOH:H_2_O 80:20, sonicated and filtered (0.45 μm). 0.2 mL of each sample was added to 2 mL of 0.1 mM DPPH solution in methanol. After an incubation at room temperature in the dark for 30 min, the absorbance was read at 517 nm. A calibration curve of Trolox (*y* = 207.91*x* − 1.8704, *R* ^2^ = 0.9966) was prepared in a range of concentrations of 0.1–0.4 mM plotting the % *I* (percentage of inhibition) against Trolox concentration. The % *I* was calculated as follows: 1.8704;

$$\%I=\frac{\mathrm{Abs}\;\text{Control}-\mathrm{Abs}\;\text{Sample}}{\mathrm{Abs}\;\text{Control}}\times 100$$

The results of each analysis, performed in triplicate, were expressed in μmol TE/g DW (TE: Trolox Equivalents).

### 2.9 ABTS Assay

The ABTS assay was performed according to Re et al. with slight modifications (Re et al. 1999). 7 mM of ABTS solution was mixed with 2.45 mM potassium persulfate (1:1) and the mixture was kept in the dark at room temperature for 12–16 h. Before starting the experiments, the ABTS solution was diluted by adding ethanol achieving an absorbance of 0.700 ± 0.02 at 734 nm. Each dried extract was solubilized in a mixture of EtOH:H_2_O 80:20, sonicated, and filtered (0.45 μm). 100 μL of each sample was mixed with 900 μL of diluted ABTS solution and incubated at room temperature in the dark for 6 min. The absorbance was read at 734 nm. The calibration curve (*y* = 656.18*x* + 0.061; *R* ^2^ = 0.9951) was prepared using Trolox as an external standard (0.025–0.125 mM) plotting the % I versus Trolox concentration. The results were expressed in μmol TE/g DW. Each analysis was performed in triplicate.

### 2.10 FRAP Assay

The FRAP assay was performed as described by Benzie et al. with slight modifications (Benzie and Strain 1996). Firstly, a 300 mM buffer acetate pH 3.6, a solution of 10 mM TPTZ in 40 mM HCl and a solution of 20 mM FeCl_3_ hexahydrate were prepared and mixed (10:1:1) to obtain the FRAP working reagent. Each dried extract was solubilized in MeOH:H_2_O 80:20, sonicated and filtered (0.45 μm). The FRAP working reagent (3 mL), kept at 37°C, was added to 100 μL of the sample. The sample was incubated in the dark for 4 min and the absorbance read at 593 nm. The calibration curve (*y* = 1.1752*x* + 0.0037, *R* ^2^ = 0.9997) was prepared using Trolox as external standard (0.05–0.9 mM) and the results were expressed in μmol TE/g DW. Each analysis was conducted in triplicate.

### 2.11 Statistical Analysis

One‐way analysis of variance (ANOVA) was applied, followed by Tukey's HSD (Honestly Significant Difference) post hoc test with a significance threshold of *p* < 0.05. In Table 1, different letters indicate statistically significant differences. HPLC and spectrophotometric analyses have been performed three times, and the results are expressed as the media of the three different experiments ± the standard deviation (SD).

**TABLE 1:** The results are reported as the media of three experiments ± the standard deviation.

Grass pea extracts	TPC (mg GAE/g DW)	TFC (mg RE/g DW)	DPPH (μmol TE/g DW)	ABTS (μmol TE/g DW)	FRAP (μmol TE/g DW)
A	2.03 ± 0.03^a^	0.28 ± 0.01^a^	na	2.78 ± 0.13^a^	na
B	2.08 ± 0.05^a^	0.27 ± 0.03^a^	na	3.07 ± 0.48^a^	na
C	1.66 ± 0.05^b^	0.23 ± 0.01^a^	2.35 ± 0.10	2.71 ± 0.21^a^	2.33 ± 0.08

*Note:* Different letters indicate statistically significant differences among the samples (*p* < 0.05).

Abbreviations: ABTS, 2,2′‐azino‐bis(3‐ethylbenzothiazoline‐6‐solfonic acid); DPPH, 2,2‐diphenyl‐1‐picrylhydrazyl; DW, dry weight of grass pea; FRAP, ferric reducing antioxidant power; GAE, gallic acid equivalents; na, not active; RE, Rutin equivalents; TE, trolox equivalents; TFC, total flavonoid content; TPC, total phenolic content.

## 3 Results and Discussion

### 3.1 β‐ODAP Profile

This work aimed to compare three Italian cultivars of grass pea and type of crop cultivation. Since the extended soaking of grass pea is able to reduce the content of β‐ODAP (Solovieva et al. 2025), water was chosen as extraction solvent, being capable of solubilizing this amino acid.

Thus, aqueous extracts of the three different cultivars of grass pea have been prepared observing the highest extraction yield for the B extract (17.9%), followed by the A extract (16.4%), and for the C extract (12.2%). The difference in extraction yields, applying the same extraction procedure, could be due to the different areas of origin, as well as different climatic and growing conditions.

In this study, a derivatization procedure using Fmoc chloride (0.2 M in acetone) has been applied following Geda et al. with slight modifications, in order to determine the β‐ODAP content in the grass pea samples by HPLC‐DAD. As previously assessed, the molar concentration of the Fmoc reagent should be at least 100 times more than the β‐ODAP concentration to complete the reaction (Geda et al. 1993).

As reported in 2.4, at the end of the process the aqueous layer was filtered after the separation from the organic layer (*n*‐hexane/ethyl acetate) and dried by rotary evaporation. Indeed, the Fmoc‐β‐ODAP results in being soluble in acetone and insoluble in *n*‐hexane and ethyl acetate, used to remove potential interferents insoluble in aqueous conditions (e.g., unreacted reagents or side products).

After the derivatization of the extracts, the aqueous solvent was removed by rotary evaporation and high vacuum to guarantee a higher stability, and the dry extracts were resolubilized in water before the HPLC analysis. To work under equal conditions, the analytical standard was treated using the same procedure. The calibration curve (*R* ^2^ > 0.998) was obtained considering eight concentrations between 5 and 1000 mg L^−1^.

**FIGURE 4:**
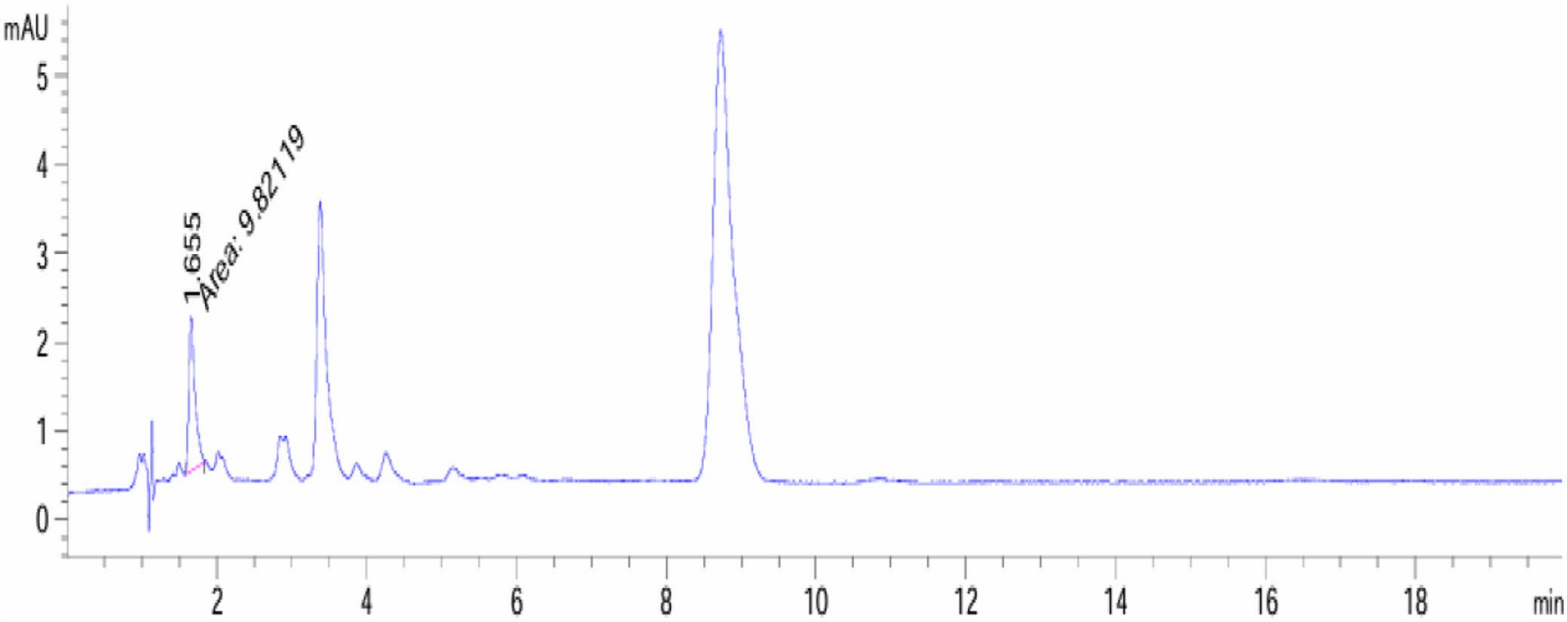
HPLC‐DAD chromatogram of Fmoc‐derivatized sample A extract.

As shown in Figure 4, the peak of Fmoc‐β‐ODAP in sample A extract has a retention time (t_R_) of 1.6 and a peak area of 9.8. The final content of β‐ODAP is 0.01 mg/g of matrix.

**FIGURE 5:**
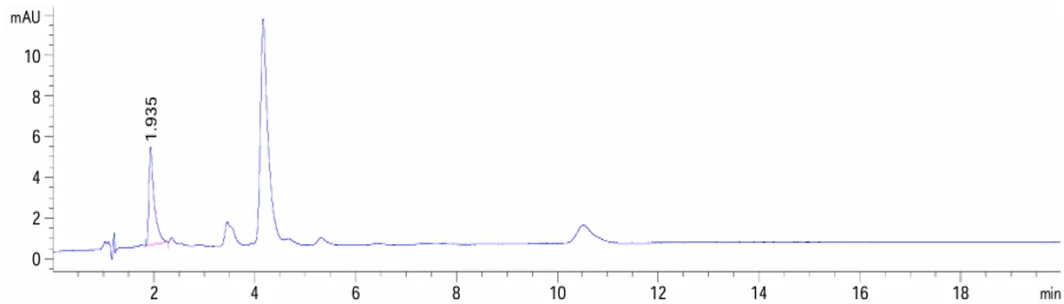
HPLC‐DAD chromatogram of Fmoc‐derivatized sample B extract.

The peak of Fmoc‐β‐ODAP in the derivatized sample B extract has t_R_ of 1.9 and a peak area of 35 (Figure 5). The measured β‐ODAP content is 0.037 mg/g.

**FIGURE 6:**
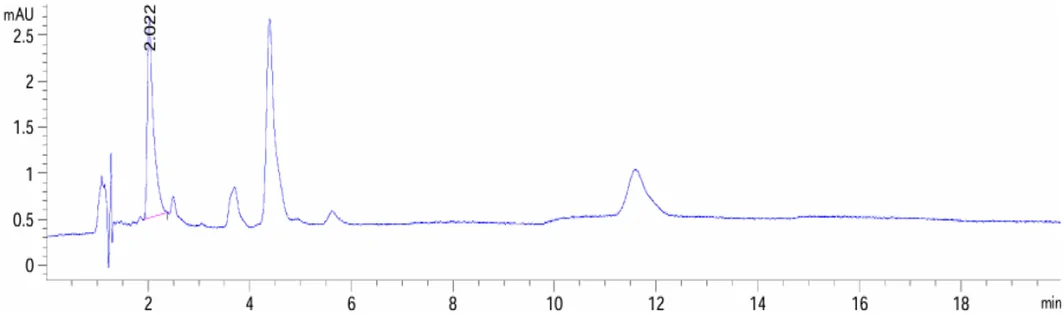
HPLC‐DAD chromatogram of Fmoc‐derivatized sample C extract.

As shown in Figure 6, the peak of Fmoc‐β‐ODAP in sample C extract has a t_R_ of 2.022 and a peak area of 18.3. The β‐ODAP content of sample C is 0.014 mg/g of matrix.

**FIGURE 7:**
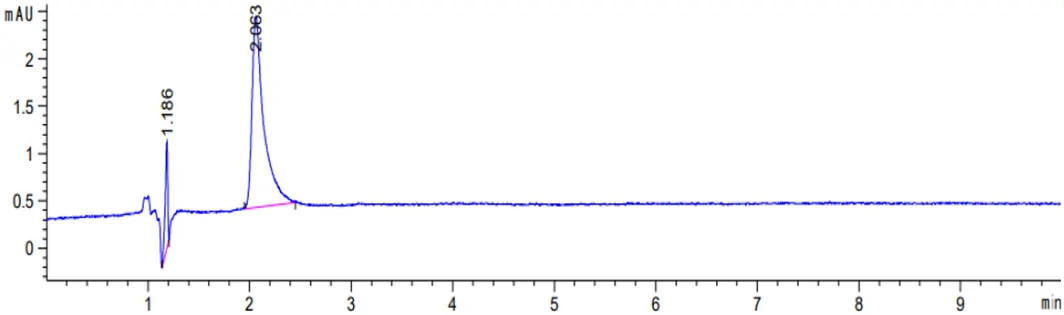
HPLC‐DAD chromatogram of Fmoc‐derivatized β‐ODAP analytical standard.

The quantification of β‐ODAP in each sample has been done in comparison with the Fmoc‐functionalized β‐ODAP standard, at the same operational conditions (Figure 7).

To summarize, the β‐ODAP content ranged from 0.01 to 0.037 mg/g of grass pea powder. The highest result was observed for the biological variety cultivated in the Umbria region. Different results have been reported in the literature regarding the levels of β‐ODAP found in grass pea; for instance, Geda et al. described a content of 0.33 g/100 g of grass pea seeds, while Tamburino et al. reported a content of 16.2 ± 0.5 g/Kg of β‐ODAP in Valle Agricola *L. sativus* seeds (Italy) (Geda et al. 1993; Tamburino et al. 2012).

Cysteine is crucial in sulfur metabolism since it contains both sulfur and nitrogen necessary for the production of essential metabolites, such as β‐ODAP (Kopriva et al. 2012). CAS enzyme catalyzes the transformation of this amino acid and cyanide to β‐cyanoalanine and H_2_S. Thus a fine regulation of cysteine biosynthesis and its degradation to H_2_S is requested in maintaining cysteine‐homeostasis, in regulating sulfide‐status and antioxidant defense of plants under stress (Alvarez et al. 2010; Xu et al. 2017). H_2_S influences seed germination, abiotic stress tolerance, and senescence, and it also competes with ROS and NO in reactions involving thiol‐containing proteins (Chen et al. 2011; Hancock et al. 2005; Hancock and Whiteman 2014; Lindermayr et al. 2005; Xu et al. 2017). This could be potentially related to the biosynthesis of β‐ODAP in grass pea, but further studies are due to elucidate this aspect. Nutritional deficiencies in sulfur‐containing amino acids such as methionine and cysteine may potentiate the neurotoxicity of β‐ODAP, since its biosynthetic pathway is linked to sulfur metabolism. Actually, few studies investigated the influence of sulfur assimilatory enzymes in grass pea and their role in β‐ODAP formation (Xu et al. 2017).

Different growing factors play a role in the changes in the β‐ODAP concentrations, indeed, the changes in its content were previously evaluated in relation with the development stage of the plant and environmental factors (e.g., drought, toxic heavy metals, nutrient deficiency and salinity) (Jiao et al. 2011). Generally, some processing techniques allow the reduction of β‐ODAP, anti‐nutritive factors and enzyme inhibitors, for example, germinating, soaking, boiling, fermentation, roasting, autoclaving or extrusion and breeding Low‐ODAP genotype (Solovieva et al. 2025).

Breeding a low‐ODAP genotype in grass pea aims to eliminate neurolathyrism while preserving the crop's drought tolerance. Traditional varieties contain 0.5%–2.5% ODAP, but breeders target levels below 0.1%. This is achieved using several primary approaches such as mutation breeding, conventional cross‐breeding, genomic tools, analytical validation (Madke et al. 2026). The values detected in our samples are even lower than those reported by the genetic improvement of grass pea through gamma‐ray‐induced mutagenesis, supporting the importance of crop and land in the development of specific breeding lines.

Overall the content of β‐ODAP in these extracts is lower than the safe level for human consumption (below 2 g.kg^−1^) and in line with other results described for grass pea seeds in Italy (Piergiovanni et al. 2011). However, further studies are due to investigate the correlation between their genotype and the influence of different environments and agricultural practices.

### 3.2 TPC, TFC, and Antioxidant Activity of Grass Pea Samples

Both non edible and edible plants represent a source of phenolic compounds which are responsible for numerous biological effects inclusive of antioxidant activity (El Gharras 2009). In this work, the total phenolic content was determined by performing the Folin–Ciocalteu assay, while the Aluminum chloride assay was applied to evaluate the total flavonoid content of each grass pea sample.

The presence of polyphenols in diverse grass pea seeds has been previously reported (Bouhrem et al. 2026; Pastor‐Cavada et al. 2009; Wang et al. 1998), but we determined their content in other cultivars. The results of these assays are in Table 1 along with those of the antioxidant assays.

Sample C shows the lowest TPC, but the best results in terms of antioxidant effect. The antioxidant properties of each sample were evaluated by applying DPPH, ABTS, and FRAP assays. DPPH and ABTS assays are radical scavenging‐based techniques, which are widely used to assess the antioxidant properties of food, beverage, and fruit and vegetable extracts. Indeed, the DPPH· and ABTS^+^ are chromogen radicals that react with the antioxidant compounds (Gulcin and Alwasel 2023). FRAP assay is a method which evaluates the ferric reducing ability of antioxidant compounds in acidic conditions (Benzie and Strain 1996). No measurable activity was observed for the A and B samples in DPPH and FRAP assays under the applied assay conditions. On the contrary, C exhibited a good radical scavenging activity both in DPPH and ABTS, as well as a reducing effect in the FRAP assay. It is interesting to note that this cultivar shows the best antioxidant profile together with a low β‐ODAP content, which is a natural antioxidant by scavenging hydroxyl ions in plants, suggesting the major influence of other bioactive compounds on the in vitro results.

## 4 Conclusion

Grass pea ( *Lathyrus sativus* L.) is a widely known crop, which is convenient to cultivate in terms of cost and ease of harvesting, being resistant to hard climatic conditions. Before post‐war period, grass pea seeds were consumed for centuries in Italy, especially in the Central and Southern Apennines (Puglia, Abruzzo and Toscana) where this crop was an accessible food source for the farmers. Its cultivation was abandoned for 50 years, but nowadays it was renewed due to the increased interest in crops typical of popular traditions and the importance of maintain a biological diversity (Lak and Almassi 2011; Tamburino et al. 2012). The nutritional profile of grass pea has been studied in terms of amino acids, fatty acids, total phenolic contents (Tamburino et al. 2012). On the other hand, the excessive human consumption of grass pea causes the ingestion of β‐ODAP, a harmful non‐protein amino acid related to the onset of neurolathyrism (Van Moorhem et al. 2011).

A comparison in terms of β‐ODAP content was conducted on three varieties of Italian grass pea to determine whether their consumption is safe for human health. The considered grass pea showed a relatively low concentration of this amino acid, which promotes its further cultivations. Nowadays, there aren't specific guidelines published by EFSA regarding the intake limits of the grass pea, but cultivars with lower content of β‐ODAP are recommended for human consumption along with the application of processing methods to reduce its content during cooking. Besides the β‐ODAP, grass pea contains also important bioactive compounds. In this work, the total phenolic and flavonoid content of each grass pea variety along with their antioxidant activity have been evaluated. Sample C, belonging to a non‐biological crop, presents the best result in terms of antioxidant activity in DPPH, ABTS, and FRAP assays, as well as having one of the lowest β‐ODAP content. Overall, these results open new perspectives in the potential of grass pea extracts for nutraceutical purposes and encourage further studies on the biofunctional properties of this old and resistant crop.

## Author Contributions

**Giulia Gentile:** investigation, methodology. **Lorenza Marinaccio:** investigation, methodology. **Eleonora Procino:** writing – original draft. **Angelo Cichelli:** funding acquisition, project administration. **Adriano Mollica:** resources, supervision. **Azzurra Stefanucci:** resources, supervision, project administration, formal analysis.

## Funding

This work was supported by NextGenerationEU.

## Ethics Statement

The authors have nothing to report.

## Conflicts of Interest

The authors declare no conflicts of interest.

## Data Availability

The data that support the findings of this study are available from the corresponding author upon reasonable request.
